# Multi-tissue DNA methylation aging clocks for sea lions, walruses and seals

**DOI:** 10.1038/s42003-023-04734-0

**Published:** 2023-04-01

**Authors:** Todd R. Robeck, Amin Haghani, Zhe Fei, Dana M. Lindemann, Jennifer Russell, Kelsey E. S. Herrick, Gisele Montano, Karen J. Steinman, Etsuko Katsumata, Joseph A. Zoller, Steve Horvath

**Affiliations:** 1 Zoological Operations, SeaWorld Parks and Entertainment, Orlando, FL USA; 2 Species Preservation Lab, SeaWorld Parks and Entertainment, San Diego, CA USA; 3 Department of Human Genetics, Gonda Research Center, David Geffen School of Medicine, Los Angeles, CA USA; 4 Altos Labs, San Diego, USA; 5 Department of Biostatistics, School of Public Health, University of California-Los Angeles, Los Angeles, CA USA; 6 SeaWorld of Florida, Orlando, FL USA; 7 SeaWorld of Texas, San Antonio, TX USA; 8 SeaWorld of California, San Diego, CA USA; 9 Kamogawa Sea World, Kamogawa, Chiba Japan

**Keywords:** DNA methylation, Conservation biology

## Abstract

Age determination of wild animals, including pinnipeds, is critical for accurate population assessment and management. For most pinnipeds, current age estimation methodologies utilize tooth or bone sectioning which makes antemortem estimations problematic. We leveraged recent advances in the development of epigenetic age estimators (epigenetic clocks) to develop highly accurate pinniped epigenetic clocks. For clock development, we applied the mammalian methylation array to profile 37,492 cytosine-guanine sites (CpGs) across highly conserved stretches of DNA in blood and skin samples (*n* = 171) from primarily three pinniped species representing the three phylogenetic families: Otariidae, Phocidae and Odobenidae. We built an elastic net model with Leave-One-Out-Cross Validation (LOOCV) and one with a Leave-One-Species-Out-Cross-Validation (LOSOCV). After identifying the top 30 CpGs, the LOOCV produced a highly correlated (r = 0.95) and accurate (median absolute error = 1.7 years) age estimation clock. The LOSOCV elastic net results indicated that blood and skin clock (*r* = 0.84) and blood (*r* = 0.88) pinniped clocks could predict age of animals from pinniped species not used for clock development to within 3.6 and 4.4 years, respectively. These epigenetic clocks provide an improved and relatively non-invasive tool to determine age in skin or blood samples from all pinniped species.

## Abstract (web summary)

DNA methylation aging clocks are generated for several species of pinnipeds enabling the estimation of pinniped age from blood or skin samples.

## Introduction

Age determination is necessary for understanding life history traits such as growth rates, age of reproductive maturity, recruitment rates, mortality rates, reproductive and somatic life span among others^1,2^. Pinnipeds are a diverse group consisting of species divided into three families, walrus (Odobenidae), sea lions (Otariidae), and seals (Phocidae), that inhabit climates ranging from sub-tropical to Artic or Antarctic regions. All species and individuals within these diverse groups are considered to play important ecological roles within their localities and are often important subsistence resources for coastal native communities globally^2,3^. In addition, the majority of species inhabit geographical areas that are predicted, with mounting real-time data, to be the most affected by climate change^4,5^. Therefore, the development of methods to assess overall individual and population health of these species is critical.

Although methods for age determination across all species have evolved toward primary reliance on cemental growth layer counting after tooth sectioning^6,7^, some of the unique morphological differences between the families have resulted in species specific methods being evaluated. For walrus, cemental tooth layer counting was first reported in the 1950s and evidence from known age, captive animals indicate high accuracy until approximately 15 years of age^8–10^. The use of mandibular bone growth layers has also been evaluated as an indicator for age assessments, but early reports of this method demonstrated conflicting results^1,11^. However, direct comparisons between teeth cemental layers and mandibular layering across a group of 70 animals found a high degree of correlation until males reached age 20 and for females up until age 10^1^. Although cemental layer age estimates appear to be accurate across a larger range of age groups within walrus, aging of older animals still becomes problematic due to poor definition of cemental layers, losses of layers due to wear and inter-observer technical variations all of which reduce accurate age estimation in older animals^12,13^.

For seals and sea lions, counting growth layer groups (GLGs) of cementum or dentine within teeth is also the primary means for determining animal age^7,14–17^. Furthermore, within some species, morphometrics in combination with other indicators (e.g., tooth eruption, canine length, cranial sutures) have also been developed to improve age estimation within these cohorts^17–21^. As with walrus and other species, difficulties in relying on teeth GLGs for age determination include loss of resolution in older animals, variability of persons performing the analysis, and invasive tooth extraction, which is typically acquired post-mortem^2,22^. Due to the general unavailability of teeth antemortem, scientists have developed non-invasive methods for age determination of animals within a herd based on the visual assessment of morphometrics^2,19^. However, within most pinniped species, morphometric estimates are generally limited to large age group classes, e.g., neonates, juvenile, and adults^8,17,20^. For walrus, additional resolution of animal ages within eight age categories has been recently demonstrated using a tusk to snout length ratio^2^. Although age category determination can be affected by quality of the photograph and angle of view, this technique shows great promise for improving our understanding of walrus herd distributions and population health. However, even at its optimum, it has not been thoroughly validated across known age adult animals, and they were not able to resolve age classes of animals beyond the age of 15. Knowing the age of these older animals is especially important for determining survivorship and fecundity, important components of all population models.

Although current aging techniques using teeth have proven accurate, they are difficult to collect antemortem and often considered too invasive for routine health exams. In addition to robust samples available from animals collected from subsistence harvests, remote biopsy and health examination of individuals are becoming important biologic resources for evaluating overall population health^23–29^. The samples from all organs, but primarily skin and blood cells, collected during these events may provide important data on the population health using epigenetic markers^30,31^. DNA methylation which refers to the transfer or removal of a methyl (CH3) group(s) from the S-adenosyl methionine (SAM) to the fifth position of the cytosine nucleotides forming 5-methylcytosine (5mC), is a well-known epigenetic change in which the magnitude of change within certain CpG sites has been demonstrated to be correlated with age (epigenetic clocks) in multiple species^31–33^. Initial attempts at developing epigenetic aging clocks in marine mammals have, to our knowledge, only been reported for cetaceans and they initially relied on the identification of methylation changes in CPGs associated with two to three sets of age responsive genes within skin samples^34–36^. With the recent development and application of the mammalian DNA methylation array, which has the potential to screen methylation changes in up to 37 K CpG sites, for use with animals, multiple epigenetic clocks which have been developed in cetaceans with improved accuracy over these initial attempts^30,37–40^.

Epigenetic aging clocks can provide benchmark measurements which may then be used to detect abnormal epigenetic age acceleration due to population level stressors^39,41^. However, prior to accelerated epigenetic age detection usage, these clocks must first be developed with healthy animals of known age. Here we used blood and skin samples collected from known age animals from six different species representing all three extant pinniped families to develop and validate two different combined pinniped epigenetic clocks: blood and skin; and blood clocks. Furthermore, we also developed family-specific blood and skin epigenetic clocks for families of pinnipeds from which sufficient sampling was available. Finally, we characterize individual cytosine-guanine sites (CpGs) that were correlated with age in the different pinniped species.

## Results

We obtained DNA methylation profiles of blood and skin samples (*n* = 171) from animals (*n* = 144) across 6 species, with all but 4 samples representing the Pacific walrus (*Odobenus rosmarus divergens*), harbor seal (*Phoca vitulina*), and California sea lion (*Zalophus californianus*) ranging in ages from zero to 41 years of age (Table 1). For these species, we had either blood or skin samples or both. An unsupervised hierarchical analysis clustered the methylation profiles primarily by tissue type (blood and skin) and, to a lesser extent, by species was used to identify conserved CpG sites to be screened for potential use in the epigenetic clock development (Supplementary Fig. 1).

**Table 1:** Demographic information of the pinniped species for which DNA methylation data were available.

‘Species	Common name	*N*^a^	No. of animals	No. of females	Captive born	Mean age	Median age	Min. age	Max age
*Eumetopias jubatus*	Steller sea lion	1	1	1	0	24.7	–	24.7	24.7
*Neophoca cinerea*	Australian sea lion	1	1	1	1	30.8	–	30.8	30.8
*Odobenus rosmarus divergens*	Pacific walrus	13 (11)	12	7	8	18.0	15.8	0	40.7
*Pagophilus groenlandicus*	Harp seal	2 (1)	1	0	0	17.2	–	17.2	17.2
*Phoca vitulina*	Harbor seal	68 (57)	58	36	55	11.0	8.2	0.5	48.0
*Zalophus californianus*	California sea lion	86 (69)	71	42	69	7.0	3.5	0.01	30.4

^a^Total number of blood and skin samples used for clock development, number of blood samples in parenthesis.

### Epigenetic aging models

**Fig. 1:**
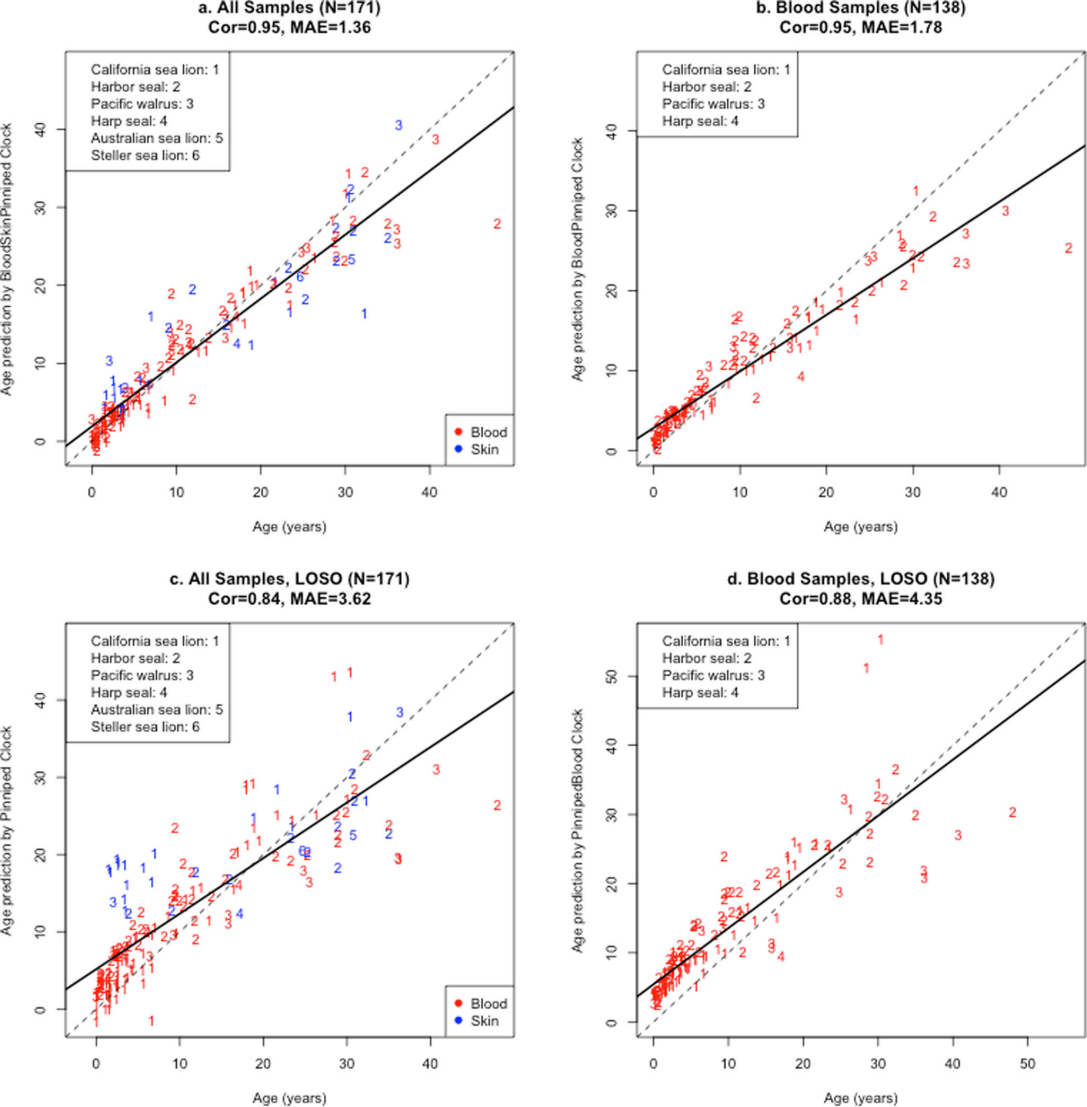
Cross-validation study of the epigenetic clock for pinnipeds. **a**, **b** report the Leave-One-Out-Cross-Validation (LOOCV) regression estimates for the pinniped clocks when applied to blood and skin (**a**) and blood only (**b**). This cross-validation method is used to estimate the clocks overall performance at predicting age from novel samples (skin or blood) collected from the animals used in clock development. **c**, **d** report Leave-One-Species-Out (LOSO) regression estimates for the pinniped clocks when applied to blood and skin (**c**) and blood only (**d**). These cross-validated analyses provide estimates for the clocks ability to predict age in species of pinnipeds that were not used for clock development. Species are presented with different integer numbers and identified in the legend; tissue types are indicated by two colors with red = blood, blue = skin. Each panel depicts a linear regression line (black dashed line), a diagonal reference line (*y* = *x*), the sample size (*N*), Pearson correlation (Cor) across all samples, and the median absolute error (MAE) across samples from all species.

We used samples from all species to create two pinniped clocks for (i) blood and skin and (ii) blood (Fig. 1). Using the Leave-One-Out-Cross-Validation (LOOCV) to assess the clocks unbiased estimates of age determination by iteratively leaving one sample out and rerunning the analysis, we found that the correlation between predicted age and chronological age, *r*, and the median absolute error (MAE) for each clock were as follows: blood and skin clock, *r* = 0.95, MAE = 1.36 years; and blood clock, *r* = 0.95, MAE = 1.78 years (Fig. 1a, b). The Leave-One-Animal-Out-Cross-Validation (LOAOCV), which leaves one animal (both skin and blood samples, if present, from an individual), produced essentially the same accuracy as the LOOCV with the results as follows: blood and skin clock, *r* = 0.94, MAE = 1.42 years; and blood clock, *r* = 0.94, MAE = 1.63 years (Supplementary Fig. 2a, b). The Leave-One-Species-Out-Cross-Validation (LOSOCV) estimates the clocks’ ability to predict age in species of pinnipeds that were not used for clock development (Fig. 1c, d). The LOSOCV results of the pinniped clock were as follows: blood and skin clock, *r* = 0.84, MAE = 3.62 years; and blood clock, *r* = 0.88, MAE = 4.35 years (Fig. 1c, d). The final versions of the pinniped clocks (based on all training data) involve 30 CpGs (blood and skin clock) and 12 CpGs (blood clock) as detailed in Supplementary Table 1. The 2 clocks share 3 CpGs in common (Supplementary Fig. 3).

**Fig. 2:**
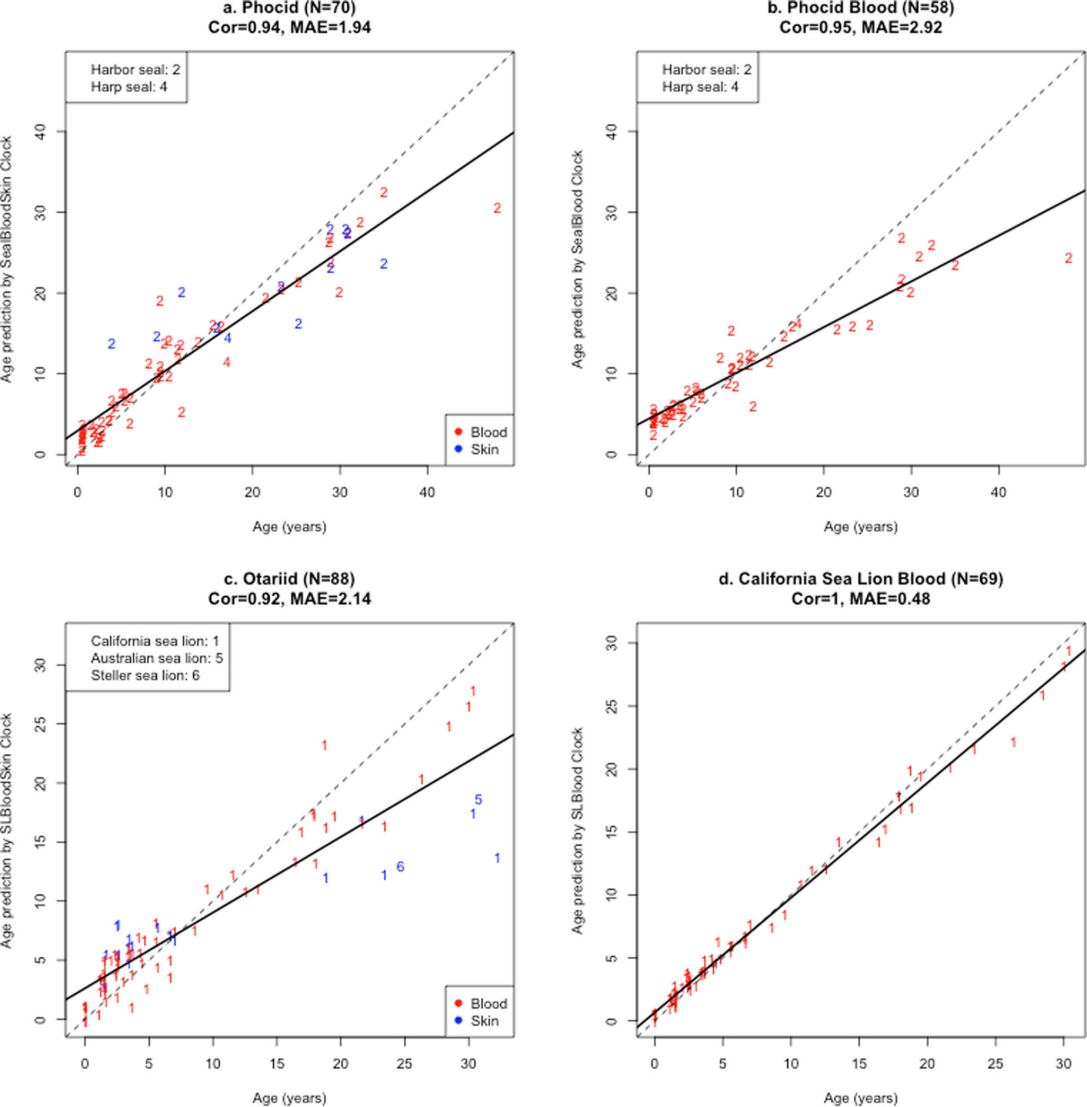
Cross-validation study of the epigenetic clock for seals (Phocidae) and sea lions (Otariidae). **a**–**d** report Leave-One-Out-Cross-Validation (LOOCV) regression estimates of the Phocid blood + skin clock (**a**), the Phocid blood clock (**b**), Otariid blood + skin clock (**c**) and California Sea Lion Blood clock (**d**). This cross-validation method was used to estimate the clocks overall performance at predicting age from novel samples (skin or blood) collected from the species used in clock development. Species are presented with different integer numbers and identified in the legend; tissue types are indicated by two colors (red = blood, blue = skin). Each panel depicts a linear regression line (black dashed line), a diagonal reference line (*y* = *x*), the sample size (*N*), Pearson correlation (Cor) across all samples, and the median absolute error (MAE) across samples from all species.

In addition to the pinniped clocks, the LOSOCV results from the pinniped family specific clocks were as follows: (i) Phocid: blood and skin clock *r* = 0.94, MAE = 1.94 years (Fig. 2a), blood clock: *r* = 0.95, MAE = 2.92 years (Fig. 2b); (ii) Otariid: blood and skin clock *r* = 0.92, MAE = 2.14 years (Fig. 2c); (iii) California Sea lion blood clock: *r* = 1.0, MAE = 0.48 years (Fig. 2d). The California sea lion blood clock was developed instead of an Otariid blood clock because we did not have any blood samples from other Otariid species.

### Epigenome-wide association studies (EWAS) of age

**Fig. 3:**
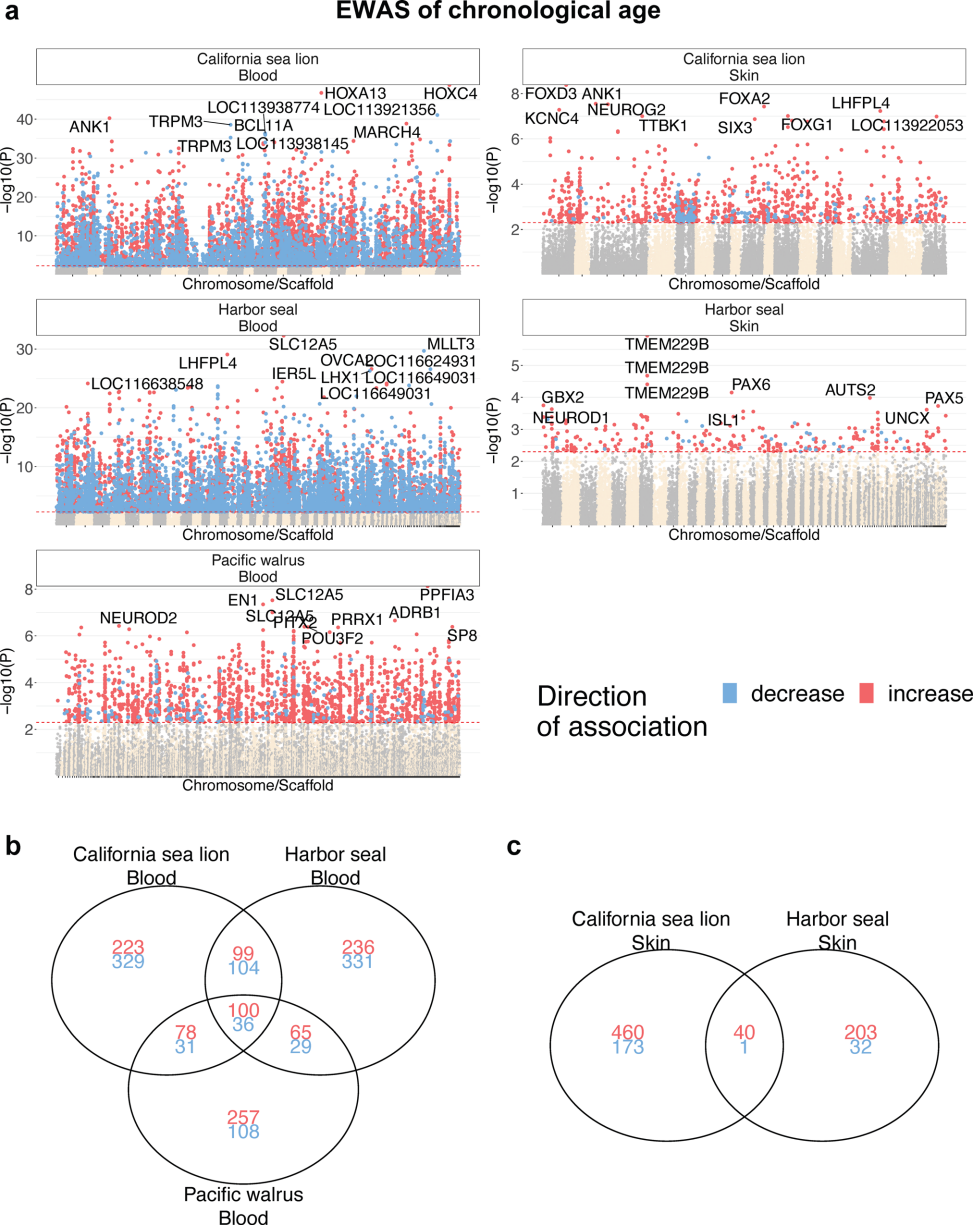
Epigenome-wide association study (EWAS) of chronological age in skin or blood of three pinniped species. **a** Manhattan plots of the EWAS of chronological age. The coordinates are based on the alignment of the probes to the corresponding genome for each species (oros_1.0, gsc_hseal_1.0, and zalcal2.2). The direction of the associations as determined by a Pearson correlation test *p* value (unadjusted *p* < 0.005, red dotted line) are colored in red (age-related increase) and blue (age-related decrease), respectively. The top 15 CpGs were labeled by the neighboring genes. **b**, **c** Venn diagrams representing the overlap of top 500 positively and top 500 negatively age-associated CpGs in each pinniped tissue. Results for all CpHs are provide in Supplementary Table 1.

In total over 31,000 probes from the 37,492 mammalian array (HorvathMammalMethylChip40) could be aligned to these three pinniped species: 31,540 in Pacific walrus, 32,075 in harbor seal, and 31,666 in California sea lion. Most of these CpGs aligned to orthologous genes, which reflected that most CpGs were located in highly conserved stretches of DNA (Supplementary Fig. 4). Many CpGs correlated significantly with age in the different pinniped tissues. At a nominal significance level of *p* < 0.005, 8148 age-related CpGs in blood from California sea lions (top CpG in HOXC4 intron); 1157 age-related CpGs in skin from California sea lions (top CpG in FOXD3 exon); 9049 age-related CpGs in blood from harbor seals (top CpG in SLC12A5 intron); 276 age-related CpGs in skin from harbor seals (top CpG in TMEM229B); and 2262 age-related CpGs in blood from Pacific walrus (top CpG in PPFIA3) were found (Fig. 3a). The difference in the number of age-related CpGs was mostly a result of differences in sample size (ranging from *n* = 11 to *n* = 69 samples) in these pinniped tissues. To control for these differences, the functional enrichment studies of CpGs were limited to the top 1000 most significant age-related CpGs (500 per direction of association) for each EWAS. The overlap analysis of these EWAS results highlighted that most of the top age-related CpGs were shared in these pinniped tissues (Fig. 3b). A subset of 5 CpGs were shared among the top 500 positively age-related CpGs in all pinniped tissues. These CpGs were located near *NEUROD1* (promoter), *PAX5* (5’UTR), *NEUROG2* (promoter), *ZIC1* (promoter), *OTP* (promoter) or the *ZIC4* (promoter) (Fig. 3b).

**Fig. 4:**
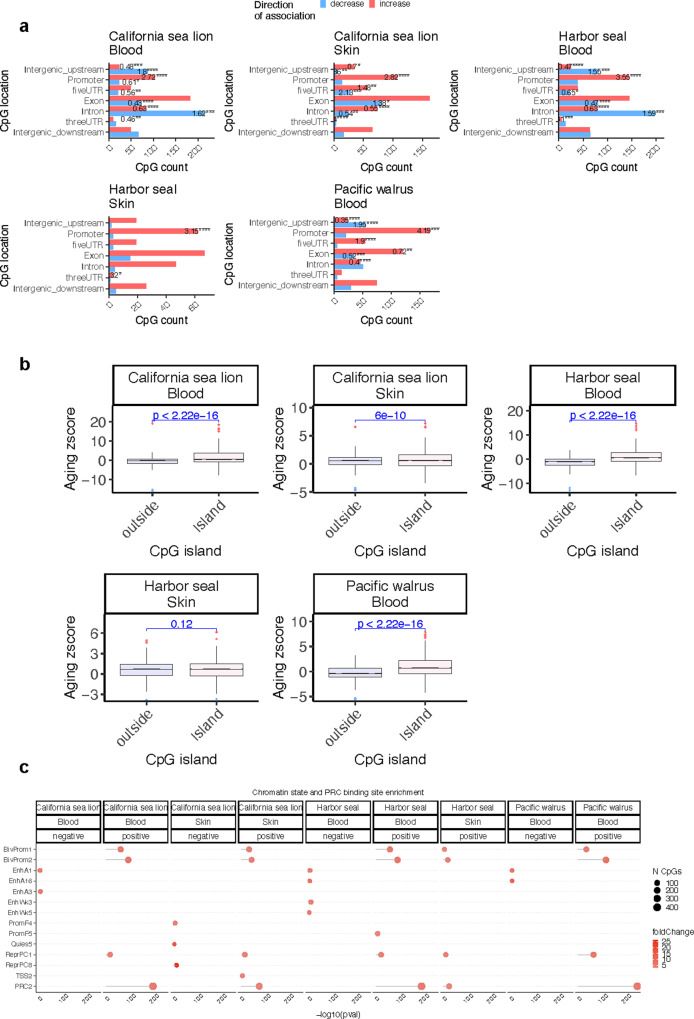
Age-related methylation changes in gene regions, CpG islands and chromatin states of pinniped species. **a** Location of top CpGs in each tissue relative to the closest transcriptional start site. The odds ratio of the observed proportional changes occurring when compared to the background are reported in each bar. Fisher exact *p* values: *p < 0.05, **p < 0.01, ***p < 0.001, ****p < 0.0001. **b** Box plot represents 25th and 75th percent quartiles, the line represents the median, and whiskers are 90% of aging association stratified by CpG island status in pinniped species. The *x* axis is the Fisher transformed *z* score of the Pearson correlation of each CpG with age. Student’s *t* test *p* values are labeled above the box plots. **c** Enrichment of the chromatic states for age-related CpGs in pinnipeds. The chromatin states are based on the StackHMM, which reports the universal chromatin states in humans^68^. The *p* values were calculated with the hypergeometric test of the EWAS results with CpGs that are in each chromatin state. The background was limited to the CpGs that could be aligned to all three pinniped genomes. The PRC2 state was defined based on the binding motif for any polycomb repressor complex 2 transcription factor (EED, SUZ12, EZH2) in human tissues.

Aging related DNAm patterns in pinnipeds were similar to those in other eutherian species^42^ (Fig. 4). These conserved patterns include an increase of methylation in promoter regions (Odds ratio >2.7, *p* < 10^−4^, Fig. 4a), a systematic gain of methylation in CpG islands (*p* < 6e-10, Fig. 4b). And finally, analysis of the chromatin states within CpGs that could be aligned between all three pinniped genomes demonstrates the close similarity of methylation patterns across several age related CpG sites between each species and within each sample matrix (Fig. 4c).

**Fig. 5:**
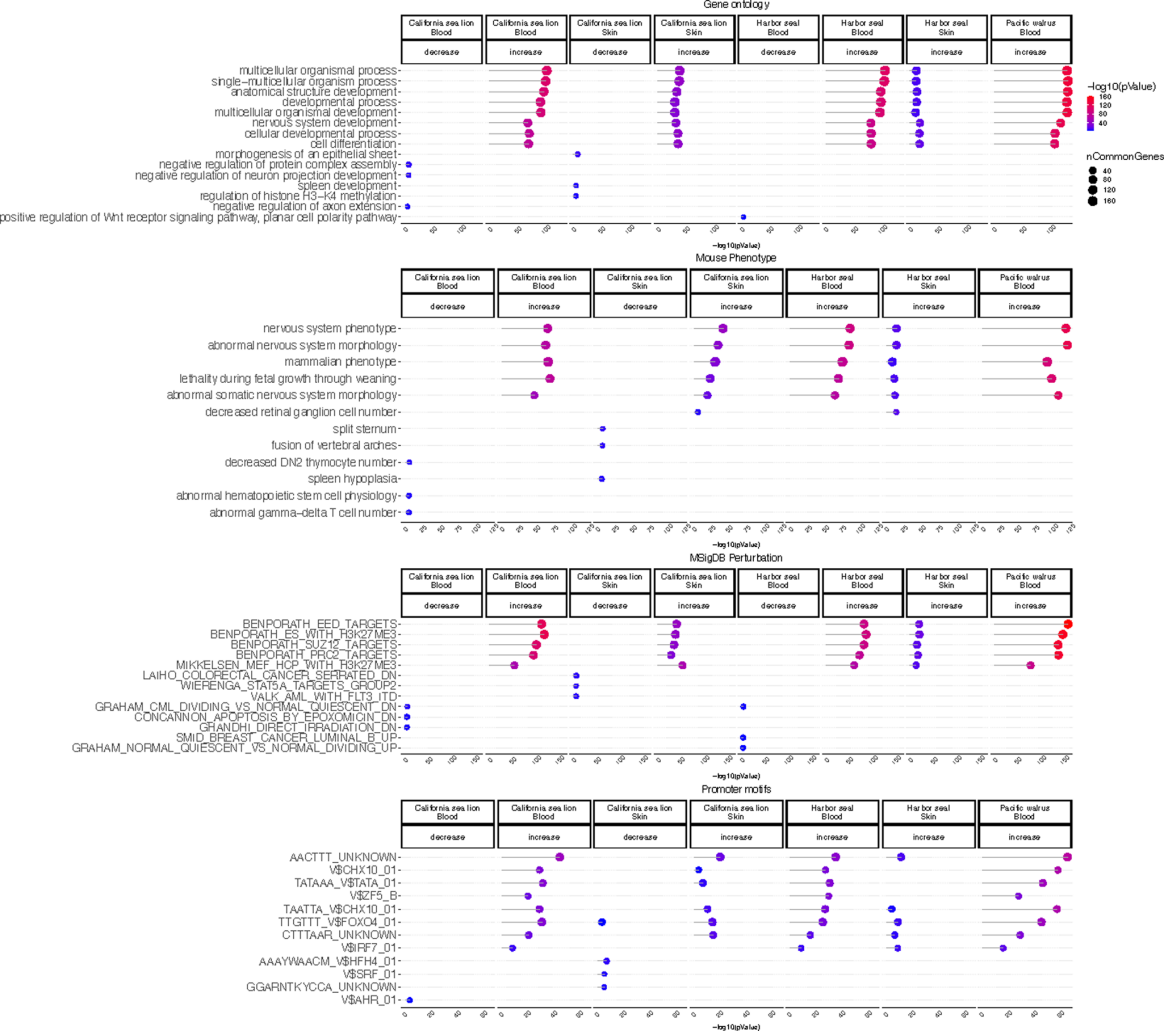
Gene set enrichment analysis of age-related CpGs in pinniped tissues. The gene level enrichment was done using GREAT analysis^69^ and human Hg19 background limited to CpGs that could be aligned to three pinniped species. The CpGs were annotated with adjacent genes in a 50 kb flanking region. We extracted up to 500 of the top CpGs based on *p* < 0.005 of association per direction of change as input for the enrichment analysis. The p values were calculated by hypergeometric test of the epigenome-wide association study (EWAS) results with the genes in each background dataset. Datasets: gene ontology; mouse phenotypes; promoter motifs; and MsigDB Perturbation, which includes the expression signatures of genetic perturbations curated in GSEA database. The results were filtered for significance at *p* < 10^−5^, minimum overlap of 3 genes, and only the top terms for each EWAS result.

A GREAT enrichment study revealed that positively age-related CpGs were located near genes that play a role in development (multicellular organismal development, morphogenesis, nervous system development, spleen development, Fig. 5) and various mouse phenotypes including (nervous system phenotype, lethality during fetal growth). In addition, overlap analysis with the Molecular Signature Data base revealed positively age-related CpGs near target sites of PRC2 (e.g., EED, SUZ12). Finally, age related CpGs were enriched in AACTTT motif (unknown transcription factor binding) and the binding sites of several transcription factors such as CHX10, ZF5_B, FOXO4 (Fig. 5).

## Discussion

This study describes the establishment of two highly accurate pinniped DNAm epigenetic aging clocks using blood or a combination of blood and skin that were developed from known age pinnipeds across their entire estimated life span. This spread of samples collected across all age groups from known age animals was critical for accurate clock development and is difficult to obtain for many species of wildlife. For the families that were sufficiently represented, Otariidae and Phocidae, we were also able to develop family specific clocks. Pinnipeds represent an extremely diverse group of animals with species that are represented in virtually all geographic locations. Despite this diversity of species and geography, over 20k CpGs were conserved across all three groups (Supplementary Fig. 2) and it was this conservation of sites that allowed us to develop a highly robust and accurate (MAE = 1.36 year, *r* = 0.95) multiple pinniped species age estimation clock. These results add to the mounting evidence from our Mammalian Methylation Consortium that indicates one can build multi-species epigenetic clocks, also known as third generation epigenetic clocks, that provide accurate age estimates across large phylogenetically diverse groups of mammals^43–45^.

Our Leave-One-Species-Out-Cross-Validation (LOSO estimate of the age correlation *r* = 0.84, median absolute error = 3.6 year) analysis demonstrated that the clock lends itself for estimating the ages of other pinniped species that were not present in the training set. However, gains in accuracy are expected to result from adding samples from other pinniped species. In practice, it is often advisable to recalibrate age predictions based on data from animals of known ages as they become available. In addition to pinniped clocks, we created blood + skin clocks that represented two of the three pinniped families (Otariidae: *r* = 0.92, MAE = 2.1 year; Phocidae *r* = 0.94, MAE = 1.94), each of which have slight improvements on the combined pinniped clock and will provide researchers improved age estimation for species within each of these families.

Although the pinniped clocks provided age estimations within 3.6 years for any pinniped species, the value of species-specific clocks cannot be overstated and is exemplified by the results from the California Sea lion blood clock which had an LOOCV generated (*r* = 1.0) MAE of 0.48 years, representing one of the most accurate epigenetic clock published to date. In addition to species specific clocks being more accurate than mixed species clocks, it has been demonstrated that multi-tissue clocks are often less accurate than tissue specific clocks with adequate numbers^30,39^. However, beyond understanding the aging process in various tissue types, it should be emphasized that the primary value for creating multi-tissue clocks in wildlife species is to increase the sample size for the analysis and thus improve its predictive value. In the case of species whereby the sample size from known age animals is limited, as is the case in this study for pinniped skin samples, this may be particularly valuable. Therefore, the creation of the blood and skin multi-species pinniped clock should prove useful for wildlife biologists who are only able to collect skin samples via remote biopsy. By combining blood and skin samples from our known age animals, and as has recently been demonstrated through the application of our odontocete clock to a novel delphinid species^40^, biologist now have a tool (blood & skin clock) to estimate an animals age to within a 1.4 years for species within this study and 3.6 years for animals from novel pinniped species.

While we did not have enough skin samples to create a pinniped skin sample only based clocks, results from other species have consistently demonstrated them to be less accurate when compared to blood clocks^30,38,39^. This is because skin samples often have higher variability in methylation rates. This variability is believed to be due to the relative differences in UV exposure of the skin at the site biopsied^46–49^. Future investigations comparing the methylation rates of different skin sampling sites within the same animal, for example along the dorsum of the animal, which is constantly exposed to UV, or along the sides with less direct exposure, may be of value.

Similar to what has been found in other species, including humans, our epigenome-wide associations (EWAS) of age in pinnipeds revealed that age-related gains in methylation were primary located in promotors, exons, and within CpG islands, while hypomethylation, or reduced methylation with age, was found with intergenic-upstream locations and introns^44,50,51^. The significance of hypomethylation is less understood, but it may be associated with the loss of regulation and control of gene expression, genomic instability, and some neoplasia^52,53^. However, unlike methylation patterns observed in cetacean skin, whereby hypomethylation across gene locations is primarily observed^39^, harbor seal and, to a lesser degree California sea lion skin samples exhibited increased methylation with age (hypermethylation) across most gene locations and no differences in methylation patterns (hypo verses hypermethylation) were detected within CpG islands. Similar to cetacean skin, relative hypomethylation with increased UV exposure as compared to sun-protected skin location has been observed in humans^47,54^. These differences may explain the apparent divergent patterns observed between cetacean (UV exposed) and pinniped skin (UV protected). However, direct comparisons of methylation patterns within each animal between sun exposed versus unexposed skin regions would be necessary to determine the exact rate of these relative changes.

Because empirical data on pinniped age-related gene expression was unavailable, we used EWAS to identify genes proximally associated with the age-related CpGs in both blood and skin. By comparing the top age-related CpGs within each species, we determined the shared age-related CpGs and genes across the three pinniped families (Phocidae, Otariidae and Odobenidae. This analysis revealed 5 genes *NEURODI, NEUROG2, PAX5, ZIC1* & *ZIC4* and *OTP* that were adjacent to CpGs with methylation patterns that increased with age. Both *NEURODI* and *NEUROG2* are important for neuronal development regulation^55,56^ as are the ZIC finger family of genes which are necessary for neurocrest development^57^. DNA methylation studies of aging in multiple species have consistently identified genes and pathways that play a role in development^51^. However, the role of age-related genes in regulating (either directly or indirectly via transcription factors) cellular proliferation, as is typically associated with tumor formation, cannot be overlooked. For example, PAX5 mutations are frequently associated with lymphoblastic leukemia^58^, and hypermethylation of ZIC1, ZIC4 or downregulation at promotor regions is associated with hepatocellular carcinoma in humans, while ZIC1 methylation is associated with gastric tumors^59,60^. Finally, loss of OTP (Orthopedia homeobox) expression, via methylation is associated with poor outcome across multiple pulmonary carcinoids^61^. Pinnipeds often acquire neoplasia^62^ compared to cetaceans that rarely present with neoplasia, unless anthropogenically induced (e.g., wild beluga^63^). Although our study was not designed to study cancer risk in different species, the patterns we observed of varying cytosine methylation levels in highly conserved stretches of DNA has the potential to yield comparative insights into species and individual animal-specific carcinogenic risks.

Our pinniped clocks provide a relatively non-invasive antemortem aging technique. These clocks can be applied to both blood and skin samples collected during routine health assessments or from skin collected from remote biopsy techniques. While methylation is affected by both genetic factors (e.g. subpopulation) and environmental confounders, with adequate sampling numbers, these confounders could give rise to an offset effect (constant difference) in the age determination which could eventually give rise to the identification of populations at risk. Therefore, future evaluation of these clocks will need to be conducted to test whether these clocks lend themselves for health assessments and the management of endangered populations of pinnipeds.

## Methods

### Ethics approval

The study was authorized by the SeaWorld Parks and Entertainment and Kamogawa Sea World’s animal care and use committees.

### Animals and sample collection

Our study included 144 animals from six species of pinnipeds with the majority being from three species, Pacific walrus (*n* = 12), California sea lion (*n* = 71) and harbor seal (*n* = 58) housed at SeaWorld of Florida (Orlando, FL), Texas (San Antonio, TX) and California (San Diego, CA) and Kamogawa Sea World (Chiba, Japan; Table 1). Animals were either captive born (*n* = 133) or orphaned as young (e.g still nursing pups) in the wild (*n* = 11) and thus based on size and seasonality of births, birth dates for wild born animals were able to be estimated to be within a few months of accuracy (Table 1).

Blood samples were collected from unrestrained animals, who had been previously conditioned for the procedure, from the hind flipper in an interdigital or metatarsal vein or from the intervertebral sinus. Blood samples were immediately placed in BD Vacutainers® containing EDTA (Becton Dickinson, Franklin Lakes, NJ), thoroughly mixed, placed into cryovials (Nunc® Cryotubes, MilliporeSigma Corp., St. Louis, MO) and frozen at −80 °C until processing. Skin samples were collected during routine health examinations under sedation. For collection, a small patch (3 ×3 cm) of hair was shaved with standard clippers just dorso-caudal to the scapula was chosen due to an abundance of excess skin and accessibility, the site was surgically prepared, and then a 6 mm biopsy punch (Integra® Miltex®, Integra, York, PA) was used to collect the sample. The incised skin was closed using veterinary surgical adhesive (Covetrus, Dublin, OH). Skin was placed in 1.2 mL cryovials (Nunc® Cryotubes) and frozen (either −20 or −80 °C) until processing.

### DNA extraction

DNA was extracted from whole blood samples using QIAamp DNA Mini blood kit and following the manufacturer’s instructions (Qiagen, Valencia, CA). Tissue samples were pulverized and broken down manually using a drill. DNA was then extracted using the automated nucleic acid extraction platform, Anaprep (Biochain, Newark, CA) that utilizes a magnetic bead extraction process and Tissue DNA Extraction kit (Anaprep).

### DNA methylation data

The extracted pinniped DNA samples were profiled using the mammalian methylation array (HorvathMammalMethylChip40) that profiles ~37k CpGs in highly conserved stretches of DNA^64^. The CpGs on the mammalian methylation array covers most genomic regions and chromatin states including CpG islands, non-islands, heterochromatin, enhancer regions, active and poised promoters, exons, bivalent promoters, transcriptional start sites. (see Fig. 3 in Arneson et al.^64^). We used this array because it facilitates comparative studies with other species. Future research could explore the utility of alternative measurement platforms that target specific CpGs underlying the presented pinniped clocks. The genomic coordinates and DNA sequences of the clock CpGs can be found in the Supplement. Out of 37,492 CpGs on the array, 35,988 probes were chosen to assess cytosine DNA methylation levels in mammalian species^64^. The subset of species for each probe can be found in the chip manifest file at Gene Expression Omnibus (GEO) at NCBI as platform GPL28271. Raw data was normalized using the SeSaMe method which assigned a beta value (0 to 1) for each methylation estimate corresponding to each probe^65^. Beta values were indicative of the proportion of chromosomes that are methylated at a given genomic location (CpG dinucleotide), with 0.5 indicating that roughly 50 percent of chromosomes are methylated.

### Pre-selection of CpGs for clock development

To ensure an unbiased assessment of predictive accuracy, the CpG selection was carried out in the training data only as previously described^39^. First, a meta-analysis using the median *Z* statistic of a correlation test was applied to the CpGs of the respective training sets to identify ~4k CpGs that had highly significant correlations with age across each species. For each CpG, a correlation test *Z* statistic for chronological age was calculated for each species-tissue stratum (blood, skin, blood and skin), and then for each training set, the strata that overlapped with the testing set were removed and the CpGs were selected based on the remaining strata.

### Clock development using penalized regression

CpGs and genome coordinates screened for clock formation and R software code used for regressions are provided in a supplementary files (Supplementary Table 1, Supplementary Date File 5). Penalized regression models were created using the R software package “glmnet”^66^ and R functions cv.glmnet, predict.glmnet. The optimal penalty parameter, Lambda, was determined in all cases by using a 10-fold internal cross-validation on the training set. Different to previous model development with cetaceans^39^, we did not transform chronological age before applying the elastic net regression model. For the regression, the elastic net penalty, Alpha, which penalizes the coefficients based on magnitude was set to ½. We used two previously described cross-validation schemes for determining unbiased estimates of the accuracy of the different DNAm aging clocks^39,42^. These were: (1) A Leave One Sample Out Cross Validation (LOOCV), (2) A leave one animal out cross validation (LOAOCV), and (3) A Leave One Species Out Cross Validation (LOSOCV) which is a modification of the LOOCV by applying it to species and instead of individual samples. Briefly, these procedures do the following for each N sample (or species): one sample is deleted from the training set (*N* − 1); which is then fit using the procedures described above and the DNAm age is predicted for the deleted sample or samples from the unused species. Therefore, the LOOCV allows us to estimate the accuracy of each sample from all species included in the training set. Due to some of the samples (one skin and one blood sample) being collected from the same animal, we also performed the LOAOCV to compare against the results from the LOOCV. The LOAOCV would assess the potential effect on the LOOCV analysis of violating the assumption of sample independence which may have been created from having skin and blood samples from the same animal. In contrast, the LOSOCV iteratively predicts the DNAm age of all samples that were removed from within a single species. This approach was used to estimate the accuracy of the pinniped clock for predicting the age of individuals within a novel species. The cross-validation procedure reports the unbiased estimates of age correlation r, which is defined as Pearson correlation between the DNAm age estimate and chronological age, as well as the median absolute error (MAE).

### Statistics and reproducibility

Data collection and statistical analysis are described in “Methods.” All statistical analyses for clock development used R Software (ver 4.0.2)^67^.

### Reporting summary

Further information on research design is available in the Nature Portfolio Reporting Summary linked to this article.

## Supplementary information

Supplementary Information

Description of Supplementary Files

Supplementary Data 1

Supplementary Data 2

Supplementary Data 3

Supplementary Data 4

Supplementary Data 5

Reporting Summary

## Data Availability

Details on the CpGs (genome coordinates) used for clock development are provided in Supplementary Table 1. Source data underlying the main figures (Figs. 1–5) are available in Supplementary Data Files 1–4. The DNA methylation data underlying this publication be found on Gene Expression Omnibus (GSE227319). Genome annotations of these CpGs can be found on Github https://github.com/shorvath/MammalianMethylationConsortium.

## Code availability

The R software code used for clock development is provided in the Supplementary Data File 5.
