# Supplementary material for: Multi-tissue DNA methylation aging clocks for sea lions, walruses and seals
Source: Commun Biol. 2023 Apr 1;6:359. doi: 10.1038/s42003-023-04734-0 (PMC10067968; doi:10.1038/s42003-023-04734-0)
Supplement: Supplementary file 1 — Supplementary Information [file 42003_2023_4734_MOESM1_ESM.pdf]

Supplementary Information 1 for:

Multi-tissue DNA methylation aging clocks for sea lions, walruses and seals

Todd R. Robeck[ǂ], Amin Haghani, Zhe Fei, Dana Lindermann, Jennifer Russell, Kelsey E.S. Herrick, Gisele Montano, Karen J. Steinman, Etsuko Katsumata, Joseph A. Zoller, Steve Horvath[ǂ]

[ǂ]*Corresponding authors*
Steve Horvath, PhD, ScD
E-mail: shorvath@mednet.ucla.edu
Todd Robeck, PhD, DVM
E-mail: todd.robeck@seaworld.com

This PDF file includes

Supplementary Table 1
Supplementary Figures 1 to 4

Supplementary Table 1. CpG sites retained after the final elastic net regression modal used for epigenetic aging clock development.

Blood and skin clock		Blood clock	
Probe ID	Model Coefficient	Probe ID	Model Coefficient
(intercept)	49.29450806	(intercept)	28.61220287
**cg00670697**	-0.33187653	**cg00764339**	30.88980172
**cg00764339**	32.32564151	**cg00930873**	6.08373985
**cg01037009**	5.56542212	**cg11728741**	25.01531776
**cg01197537**	1.56362498	**cg12017700**	123.80526190
**cg03045876**	11.26912123	**cg12841266**	4.68739715
**cg03740978**	4.92465335	**cg14870509**	-0.33840083
**cg04131501**	215.39064654	**cg16702105**	5.82507567
**cg05551621**	9.08725355	**cg18992104**	-31.90455582
**cg05897263**	18.38856672	**cg21801378**	17.84724510
**cg06031930**	-13.18142456	**cg21852818**	0.33912659
**cg06441752**	0.00225073	**cg26051365**	-1.85334983
**cg06857737**	-0.70119280	**cg26053530**	-6.91527501
**cg07254032**	26.51153603		
**cg09802094**	22.48682126		
**cg10718793**	-2.21719466		
**cg12017700**	52.60637937		
**cg12372060**	1.38066714		
**cg13433256**	15.46065164		
**cg16111936**	39.59544715		
**cg16702105**	5.15800040		
**cg16723516**	-15.37020581		
**cg20706331**	-21.44630135		
**cg21222571**	1.49816996		
**cg21476366**	1.64242819		
**cg22793142**	-39.01544948		
**cg24283073**	-0.57767193		
**cg24466972**	-2.92934308		
**cg25148589**	12.33239180		
**cg25877801**	-14.63448619		
**cg27193252**	20.07319692		

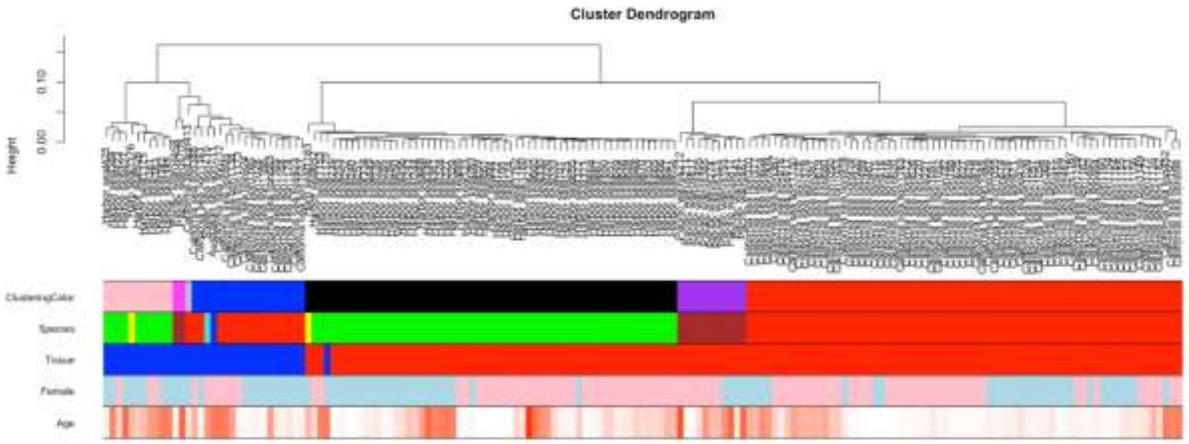

Supplementary Fig. 1 Unsupervised hierarchical clustering of pinniped samples. Average linkage hierarchical clustering based on the interarray correlation coefficient (Pearson correlation). The clustering colors (first color band) largely correspond to species (second color band) and tissue type (third color band). The individual leaves of the tree are labeled by species common names followed by the sample number: California sea lion (*Zalophus californianus*); Harbor seal (*Phoca vitulina*); Pacific walrus (*Odobenus rosmarus divergens*); Harp seal (*Pagophilus groenlandicus*); Australian sea lion (*Neophoca cinerea)*; Steller sea lion (*Eumetopias jubatus*). Explanation of the first color band: the red cluster corresponds to blood samples from California sea lions; dark red = blood from Pacific walrus; black = blood from Harbor seals and Harp seal; blue = skin from California sea lions; pink = skin from Harbor seals and Harp seal; mauve = skin from Pacific walrus.

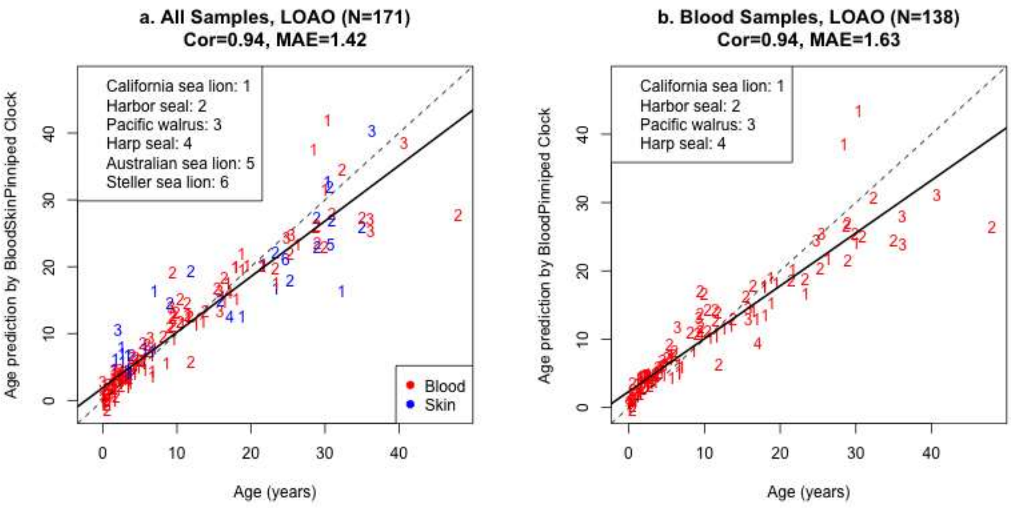

Supplementary Fig. 2. Cross-validation study of the epigenetic clock for pinnipeds. Panels a and b report the Leave-One-Animal-Cross-Validation (LOAOCV) regression estimates for the pinniped clocks when applied to blood and skin (a) and blood only (b). This cross-validation method is used to estimate the clocks overall performance at predicting age from novel animals (skin samples, blood samples or both) used in clock development. Species are presented with different integer numbers and identified in the legend; tissue types are indicated by two colors with red = blood, blue = skin. Each panel depicts a linear regression line (black dashed line), a diagonal reference line (y = x), the sample size (N), Pearson correlation (Cor) across all samples, and the median absolute error (MAE) across samples from all animals.

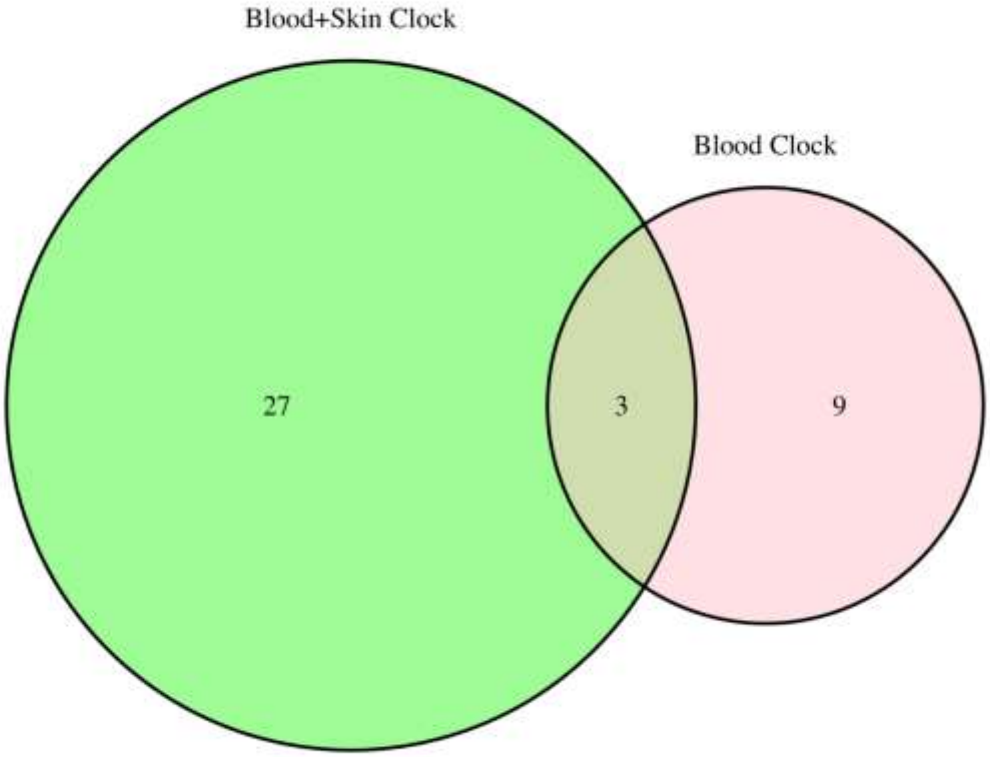

Supplementary Fig. 3. **CpGs overlap in two pinniped Clocks**

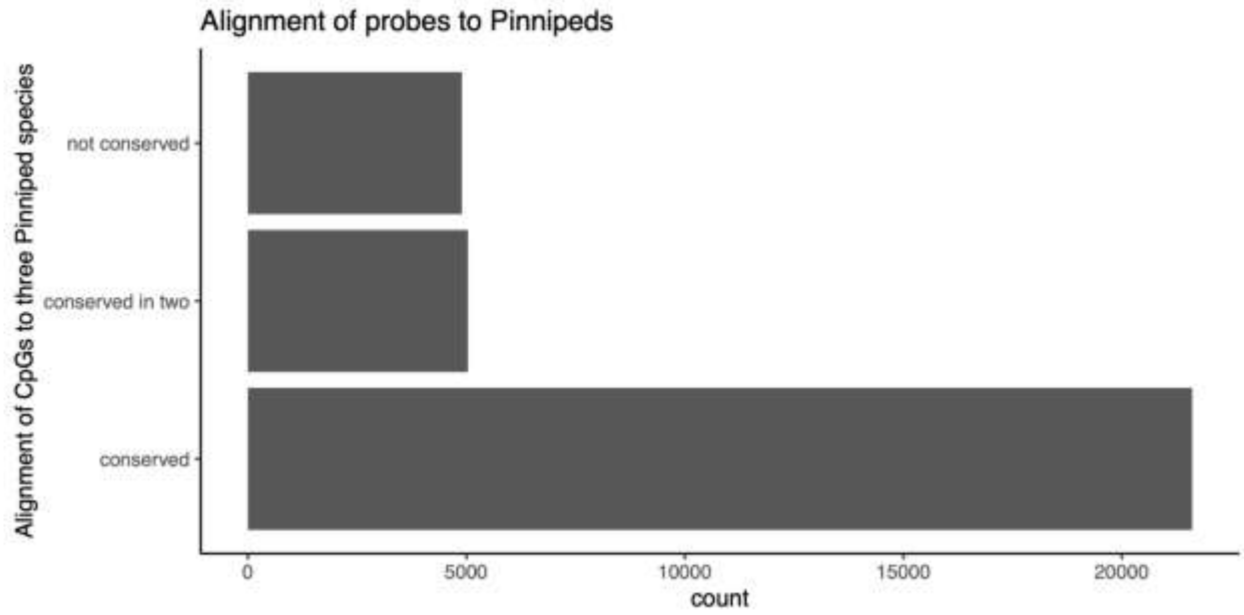

Supplementary Fig. 4. **Comparison of mammalian array coverage in three pinniped species**. Genome assemblies: Odobenus_rosmarus.oros1.0; Hoca_vitulina.gsc_hseal_1.0; Zalophus_californianus.zalcal2.2. Categories: “Conserved”, aligned to the same gene in three genomes; “conserved in two”, aligned to the same gene in two of the genomes; “not conserved”, aligned to different genes.
