# Supplementary material for: Multi-tissue DNA methylation aging clocks for sea lions, walruses and seals
Source: Commun Biol. 2023 Apr 1;6:359. doi: 10.1038/s42003-023-04734-0 (PMC10067968; doi:10.1038/s42003-023-04734-0)
Supplement: Supplementary file 2 — Description of Supplementary Files [file 42003_2023_4734_MOESM2_ESM.pdf]

# Description of Additional Supplementary Files 2

**File name:** Supplementary Data 1

**Description:** The underlying source data for Figure 1 and Figure 2.

**File name:** Supplementary Data 2

**Description:** The underlying source data for Figure 3.

**File name:** Supplementary Data 3

**Description:** The underlying source data for Figure 4.

**File name:** Supplementary Data 4

**Description:** The underlying source data for Figure 5.

**File name:** Supplementary Data 5

**Description:** R code for pinniped clock analysis and graphing
