# Supplementary material for: Multi-tissue DNA methylation aging clocks for sea lions, walruses and seals
Source: Commun Biol. 2023 Apr 1;6:359. doi: 10.1038/s42003-023-04734-0 (PMC10067968; doi:10.1038/s42003-023-04734-0)
Supplement: Supplementary file 7 — Supplementary Data 5 [file 42003_2023_4734_MOESM7_ESM.pdf]

Supplementary Information 2 for:
Multi-tissue DNA methylation aging clocks for pinnipeds: sea lions, walrus, and seals

Todd R. Robeck‡, Amin Haghani, Zhe Fei, Dana Lindermann, Jennifer Russell, Kelsey E.S. Herrick, Gisele Montano, Karen J. Steinman, Etsuko Katsumata, Joseph A. Zoller, Steve Horvath‡

‡*Corresponding authors*
Steve Horvath, PhD, ScD
E-mail: shorvath@mednet.ucla.edu
Todd Robeck, PhD, DVM
E-mail: todd.robeck@seaworld.com

This PDF file includes
R Code for pinniped clock

A) Fit the clocks

```
dat1 = read.csv("dat_pinniped_Aug16_2022.csv",row.names = 1)

######### training estimates
train.idx = which(dat1$CanBeUsedForAgingStudies=="yes"&
                dat1$ConfidenceInAgeEstimate>= 90)
y.train = dat1$Age[train.idx]
# y.train = log(dat1$Age[train.idx] + 1)
# y.train = dat1$logliAge[train.idx]
cg.idx = match(dat1$Basename[train.idx], colnames(dat0sesame))
summary(cg.idx )
length(detectP_id)

fit1 = cv.glmnet(t(dat0sesame[detectP_id,cg.idx]), y.train)
plot(fit1)
lam1 = fit1$lambda.1se
coef1 = coef(fit1, s = lam1)
coef1 = as.data.frame(coef1[coef1[,1]!= 0,])
colnames(coef1) = "Coefficient"

write.csv(coef1, "pinniped_V4/Pinniped Clock.csv")
# lam1 = fit1$lambda.min

cg.idx = match(dat1$Basename, colnames(dat0sesame))
predAge = predict(fit1, t(dat0sesame[detectP_id,cg.idx]), s=lam1)
verboseScatterplot(dat1$Age, predAge)
verboseScatterplot(dat1$Age[-train.idx], predAge[-train.idx])

dat1$DNAmAge_BloodSkinPinniped = NA
dat1$DNAmAge_BloodSkinPinniped = predAge

idx1 = which(dat1$SpeciesLatinName %in% names(speciestab)[1:3])
x = dat1$Age[idx1]
y = dat1$DNAmAge_BloodSkinPinniped[idx1]
verboseScatterplot(x,y)
abline(0,1)
######### Blood clock

train.idx = which(dat1$CanBeUsedForAgingStudies=="yes" &
                dat1$ConfidenceInAgeEstimate>= 90 &
```

```
                 dat1$Tissue == "Blood")
test.idx = which(dat1$CanBeUsedForAgingStudies=="yes" &
                 dat1$ConfidenceInAgeEstimate>= 90 &
                 dat1$Tissue == "Skin")
y.train = dat1$Age[train.idx]
# y.train = log(dat1$Age[train.idx] + 1)
# y.train = dat1$logliAge[train.idx]
cg.idx = match(dat1$Basename[train.idx], colnames(dat0sesame))

fit1 = cv.glmnet(t(dat0sesame[detectP_id,cg.idx]), y.train)
plot(fit1)
# lam1 = fit1$lambda.min
lam1 = fit1$lambda.1se
coef1 = coef(fit1, s = lam1)
coef1 = as.data.frame(coef1[coef1[,1]!= 0,])
colnames(coef1) = "Coefficient"

write.csv(coef1, "pinniped_V4/Pinniped Blood Clock.csv")

{
  coef1 = read.csv("pinniped_V4/Pinniped Clock.csv",row.names = 1)
  coef2 = read.csv("pinniped_V4/Pinniped Blood Clock.csv",row.names = 1)

  intersect(rownames(coef1), rownames(coef2))

  library(VennDiagram)
  set1 = rownames(coef1)[-1]
  set2 = rownames(coef2)[-1]
  venn.diagram(list(set1,set2),
            category.names = c("Blood+Skin Clock" , "Blood Clock"),
            filename = paste0(outfolder,'/pinnipedClocks.tiff'),
            fill= c("green","pink"),
            cat.pos = c(0,0),
            output=TRUE)
}

cg.idx = match(dat1$Basename, colnames(dat0sesame))
predAge = predict(fit1, t(dat0sesame[detectP_id,cg.idx]), s=lam1)
verboseScatterplot(dat1$Age, predAge)

dat1$DNAmAge_BloodPinniped = NA
dat1$DNAmAge_BloodPinniped = predAge

verboseScatterplot(dat1$Age[test.idx], predAge[test.idx])
```

```
colnames(dat1)
write.csv(dat1, paste0(outfolder,"/dat_pinniped.csv"))

table(dat1$Tissue)

##### LOO
for (ik in 1:2) {
  print(ik)

  train.idx = switch (ik,
                      which(dat1$CanBeUsedForAgingStudies=="yes" &
                              dat1$ConfidenceInAgeEstimate>= 90),
                      which(dat1$CanBeUsedForAgingStudies=="yes" &
                              dat1$ConfidenceInAgeEstimate>= 90 & dat1$Tissue=="Blood"))
  y.train = dat1$Age[train.idx]
  cg.idx = match(dat1$Basename[train.idx], colnames(dat0sesame))

  fit1 = cv.glmnet(t(dat0sesame[detectP_id,cg.idx]), y.train)
  plot(fit1)
  lam1 = fit1$lambda.1se
  # lam1 = fit1$lambda.min

  dat1$DNAmAge_LOO = NA
  for (i in 1:nrow(dat1)) {
    train.idx = switch (ik,
                        which(dat1$CanBeUsedForAgingStudies=="yes" &
                                dat1$ConfidenceInAgeEstimate>= 90),
                        which(dat1$CanBeUsedForAgingStudies=="yes" &
                                dat1$ConfidenceInAgeEstimate>= 90 & dat1$Tissue=="Blood"))
    train.idx = setdiff(train.idx, i)
    y.train = dat1$Age[train.idx]

    cg.idx = match(dat1$Basename[train.idx], colnames(dat0sesame))
    test.cg = match(dat1$Basename[i], colnames(dat0sesame))

    fit1 = glmnet(t(dat0sesame[detectP_id,cg.idx]), y.train, lambda = lam1)

    # predAge = predict(fit1, t(dat0sesame[detectP_id,test.cg]), s="lambda.min")
    predAge = predict(fit1, t(dat0sesame[detectP_id,test.cg]))
    dat1$DNAmAge_LOO[i] = predAge ## exp(predAge)-1 ###revtrsf(predAge, m1[i])
```

```
    # print(i)
  }
  summary(dat1$DNAmAge_LOO)


  train.idx = switch (ik,
                    which(dat1$CanBeUsedForAgingStudies=="yes" &
                            dat1$ConfidenceInAgeEstimate>= 90),
                    which(dat1$CanBeUsedForAgingStudies=="yes" &
                            dat1$ConfidenceInAgeEstimate>= 90 & dat1$Tissue=="Blood"))
  x = dat1$Age[train.idx]
  y = dat1$DNAmAge_LOO[train.idx]
  # y = dat1$DNAmAge_BloodSkin_LOO[train.idx]
  verboseScatterplot(x, y, type="n")
  text(x,y, dat1$OriginalOrderInBatch[train.idx])
  abline(0,1)

  # colnames(dat1)[55] = "DNAmAge_BloodSkinPinniped_LOO"
  colnames(dat1)[grep("DNAmAge_LOO", colnames(dat1))] =
    paste0(colnames(dat1)[52+ik], "_LOO")

}

colnames(dat1)
write.csv(dat1, paste0(outfolder,"/dat_pinniped.csv"))
# dat1 = read.csv(paste0(outfolder,"/dat_pinniped_June8.csv"), row.names = 1)


######### single species clock
with(dat1, table(ConfidenceInAgeEstimate, CanBeUsedForAgingStudies))


colnames(dat1)


### single species clocks
ik = 5
for (ik in 2:5) {
  agename = switch (ik,
                  "_bloodage",
                  "_SealBloodClock",
                  "_SLBloodClock",
                  "_SealBloodSkinClock",
                  "_SLBloodSkinClock")
```

```
specname = switch(ik-1,
              c("Harbor seal", "Harp seal"),
              "California sea lion",
              "Harbor seal",
              "California sea lion")
if(ik==1) specidx = which(dat1$CanBeUsedForAgingStudies=="yes" &
                     dat1$ConfidenceInAgeEstimate>= 90) else specidx =
  which(dat1$CanBeUsedForAgingStudies=="yes" &
       dat1$ConfidenceInAgeEstimate>= 90 & dat1$SpeciesCommonName %in% specname)


if(ik<= 3) {
  train.idx = intersect(which(dat1$Tissue == "Blood"), specidx)
  test.idx = intersect(which(dat1$Tissue == "Skin"), specidx)

}else train.idx = test.idx = specidx


y.train = dat1$Age[train.idx]
cg.idx = match(dat1$Basename[train.idx], colnames(dat0sesame))
test.cg = match(dat1$Basename[test.idx], colnames(dat0sesame))


fit0 = cv.glmnet(t(dat0sesame[detectP_id,cg.idx]), y.train)
plot(fit0)
lam1 = fit0$lambda.1se
# lam1 = fit0$lambda.min

# test.idx = which(dat1$SpeciesNumber==4)
# test.cg = match(dat1$Basename[test.idx], colnames(dat0sesame))

predAge = predict(fit0, t(dat0sesame[detectP_id,test.cg]), s=lam1)
x = dat1$Age[test.idx]
y = predAge
verboseScatterplot(x, y)
abline(0,1)

##### LOO
# dat1$DNAmAge_age_LOO = NA
dat1$DNAmAge_LOO = NA
for (i in 1:nrow(dat1)) {
  if(ik<= 3) train.idx = intersect(which(dat1$Tissue == "Blood"), specidx) else
    train.idx = specidx
```

```
    train.idx = setdiff(train.idx, i)
    y.train = dat1$Age[train.idx]

    cg.idx = match(dat1$Basename[train.idx], colnames(dat0sesame))
    test.cg = match(dat1$Basename[i], colnames(dat0sesame))

    fit1 = glmnet(t(dat0sesame[detectP_id,cg.idx]), y.train, lambda = lam1)

    predAge = predict(fit1, t(dat0sesame[detectP_id,test.cg]))
    dat1$DNAmAge_LOO[i] = predAge

    #print(i)
  }
  print(i)
  verboseScatterplot(dat1$Age[train.idx], dat1$DNAmAge_LOO[train.idx])
  agename

  if(FALSE){
    train.idx = intersect(which(dat1$Tissue == "Blood"), specidx)
    train.idx = intersect(which(dat1$Tissue == "Skin"), specidx)
    train.idx = specidx
    x = dat1$Age[train.idx]
    y = dat1$DNAmAge_LOO[train.idx]
    verboseScatterplot(x, y)
    abline(0,1)

  }

  colnames(dat1)[grep("DNAmAge_LOO", colnames(dat1))] =
paste0("DNAmAge",agename,"_LOO")


}

colnames(dat1)
speciestab
dat1$SpeciesNumber = NA
for (k in length(speciestab):1) {
  dat1$SpeciesNumber[dat1$SpeciesLatinName ==
                     names(speciestab)[k]] =
    length(speciestab)+1-k
}
table(dat1$SpeciesNumber)
```

```
write.csv(dat1, paste0(outfolder,"/dat_pinniped.csv"))


## B) LOSO Estimates


######### training estimates
y.train = dat1$Age[train.idx]

cg.idx = match(dat1$Basename[train.idx], colnames(dat0sesame))

fit1 = cv.glmnet(t(dat0sesame[detectP_id,cg.idx]), y.train)
plot(fit1)
lam1 = fit1$lambda.1se

tabspecs = table(dat1$SpeciesNumber)
train.all = which(dat1$CanBeUsedForAgingStudies=="yes"&
                    dat1$ConfidenceInAgeEstimate>= 90)

dat1$DNAmAge_LOSO = NA
for (i in 1:length(tabspecs)) {

  test.idx = specidx = which(dat1$SpeciesNumber == i)
  train.idx = setdiff(train.all, test.idx)

  y.train = dat1$Age[train.idx]
  cg.idx = match(dat1$Basename[train.idx], colnames(dat0sesame))
  test.cg = match(dat1$Basename[test.idx], colnames(dat0sesame))

  fit1 = cv.glmnet(t(dat0sesame[detectP_id,cg.idx]), y.train )

  predAge = predict(fit1, t(dat0sesame[detectP_id,test.cg]))
  dat1$DNAmAge_LOSO[test.idx] = predAge ## exp(predAge)-1 ###revtrsf(predAge, m1[i])

  # print(i)
}
summary(dat1$DNAmAge_LOSO)

nc1 = 63
colnames(dat1)[nc1] = "DNAmAge_PinnipedClock_LOSO"

### Blood LOSO
{
  train.all = which(dat1$CanBeUsedForAgingStudies=="yes"&
```

```
              dat1$ConfidenceInAgeEstimate>= 90 &
              dat1$Tissue == "Blood")
  tabspecs = table(dat1$SpeciesNumber[train.all])

  dat1$DNAmAge_LOSO = NA
  for (i in 1:length(tabspecs)) {

    test.idx = specidx = intersect(which(dat1$SpeciesNumber == i), train.all)
    train.idx = setdiff(train.all, test.idx)

    y.train = dat1$Age[train.idx]
    cg.idx = match(dat1$Basename[train.idx], colnames(dat0sesame))
    test.cg = match(dat1$Basename[test.idx], colnames(dat0sesame))

    fit1 = cv.glmnet(t(dat0sesame[detectP_id,cg.idx]), y.train )

    predAge = predict(fit1, t(dat0sesame[detectP_id,test.cg]))
    dat1$DNAmAge_LOSO[test.idx] = predAge ## exp(predAge)-1 ###revtrsf(predAge, m1[i])

    # print(i)
  }
  summary(dat1$DNAmAge_LOSO)

  x = dat1$Age[train.all]
  y = dat1$DNAmAge_LOSO[train.all]
  plot(x, y)
  abline(0,1)

}

colnames(dat1)
nc1 = 64
colnames(dat1)[nc1] = "DNAmAge_PinnipedBloodClock_LOSO"

tiff(paste0(outfolder,"/PinnipedClocks_LOSO_v2.tiff"),
    width = 800,height = 400)
par(mfrow=c(1,2),mar = c(5, 5, 4, 2) + 0.1)
{
  nc1 = 63
  train.idx = which(dat1$CanBeUsedForAgingStudies=="yes"&
              dat1$ConfidenceInAgeEstimate>= 90)
  plotfn(dat1, nc1, train.idx, "a. All Samples, LOSO",
       includelegend=FALSE)
  legend("bottomright",c("Blood","Skin"),
```

```
       pch = 19, col = c("red","blue"))
  legend("topleft",bg="transparent",
       c("California sea lion: 1",
                  "Harbor seal: 2",
                  "Pacific walrus: 3",
                  "Harp seal: 4",
                  "Australian sea lion: 5",
                  "Steller sea lion: 6"))

  nc1 = 64
  train.idx = which(dat1$CanBeUsedForAgingStudies=="yes"&
                   dat1$ConfidenceInAgeEstimate>= 90 &
                   dat1$Tissue == "Blood")
  plotfn(dat1, nc1, train.idx, "b. Blood Samples, LOSO",
       includelegend=FALSE)
  legend("topleft",bg="transparent",
       c("California sea lion: 1",
                  "Harbor seal: 2",
                  "Pacific walrus: 3",
                  "Harp seal: 4"))

}
dev.off()


write.csv(dat1, paste0(outfolder,"/dat_pinniped_Aug16_2022.csv") )


## C) Plots

plotfn = function(dat1, nc, idx, name1, includelegend=FALSE, tissuecolor = TRUE){
  ylab = strsplit(colnames(dat1)[nc],"_")[[1]][2]
  x = dat1$Age[idx]
  y = dat1[idx, nc]
  xr = range(x,y, na.rm = T)

  mae = round(median(abs(x-y)), 2)
  cor1 = round(cor(x,y),2)

  plot(x, y, type="n", xlab = paste0("Age (N=",length(x),")"),
                  ylab = ylab,
                  main = paste0(name1,"\nCor=",cor1,", MAE=",mae),
                  xlim=xr, ylim=xr,)
  if(tissuecolor){
```

```
    text(x,y, dat1$SpeciesNumber[idx], col = dat1$TissueColor[idx])
    if(includelegend) legend("topleft",c("Blood","Skin"),
                             pch = 19, col = c("red","blue"))

  }else{
    text(x,y, dat1$SpeciesNumber[idx], col = dat1$SpeciesColor[idx])
    if(includelegend) legend("topleft",
                             c("Harbor seal","California sea lion","Pacific walrus"),
                             pch = 19, col = c("firebrick4","maroon","bisque4"))

  }
  abline(0,1, lty=2)
  abline(coef(lm(y~x)), lwd=2)

}

# dat1 = read.csv("pinnedV3/dat_pinniped_Dec1.csv",row.names=1)

outfolder = "pinniped_V4"
write.csv(dat1, paste0(outfolder,"/dat_pinniped_Mar2_2022.csv") )

pdf(paste0(outfolder,"/Pinniped_bloodclock_v3.pdf"), onefile = TRUE)
par(mfrow=c(2,2))
#####Pinniped Clocks
{
  nc1 = 53
  train.idx = which(dat1$ConfidenceInAgeEstimate>= 90)
  test.idx = which(dat1$ConfidenceInAgeEstimate< 90)

  plotfn(dat1, nc1, train.idx, "AllTrainingSamples",
         includelegend=TRUE,
         tissuecolor = TRUE)
  plotfn(dat1, nc1, test.idx, "LowConfidenceSamples",
         includelegend=FALSE,
         tissuecolor = TRUE)



  nc=54
  train.idx = which(dat1$ConfidenceInAgeEstimate>= 90 & dat1$Tissue == "Blood")
  test.idx = which(dat1$ConfidenceInAgeEstimate>= 90 & dat1$Tissue == "Skin")

  plotfn(dat1, nc, train.idx, "BloodSamples",
         includelegend=TRUE, tissuecolor = FALSE)
  plotfn(dat1, nc, test.idx, "SkinSamples",
```

```
      includelegend=FALSE,tissuecolor = FALSE)


  nc1 = 55
  train.idx = which(dat1$ConfidenceInAgeEstimate>= 90)
  test.idx = which(dat1$ConfidenceInAgeEstimate< 90)

  plotfn(dat1, nc1, train.idx, "AllSamples, LOO",
      includelegend=TRUE,
      tissuecolor = TRUE)


  nc=56
  train.idx = which(dat1$ConfidenceInAgeEstimate>= 90 & dat1$Tissue == "Blood")
  test.idx = which(dat1$ConfidenceInAgeEstimate>= 90 & dat1$Tissue == "Skin")

  plotfn(dat1, nc, train.idx, "BloodSamples, LOO",
      includelegend=TRUE, tissuecolor = FALSE)

}
#####Single Species LOO
{
  nc1 = 59
  train.idx = which(dat1$ConfidenceInAgeEstimate>= 90&
            dat1$SpeciesCommonName == "Harbor seal")
  # test.idx = which(dat1$ConfidenceInAgeEstimate< 90)

  plotfn(dat1, nc1, train.idx, "Harbor seal, LOO",
      includelegend=TRUE,
      tissuecolor = TRUE)


  nc=57
  train.idx = which(dat1$ConfidenceInAgeEstimate>= 90 & dat1$Tissue == "Blood"&
            dat1$SpeciesCommonName == "Harbor seal")

  plotfn(dat1, nc, train.idx, "Harbor seal, Blood, LOO",
      includelegend=TRUE, tissuecolor = FALSE)



  nc1 = 60
  train.idx = which(dat1$ConfidenceInAgeEstimate>= 90&
            dat1$SpeciesCommonName == "California sea lion")
```

```
  # test.idx = which(dat1$ConfidenceInAgeEstimate< 90)

  plotfn(dat1, nc1, train.idx, "California sea lion, LOO",
       includelegend=TRUE,
       tissuecolor = TRUE)



  nc=59
  train.idx = which(dat1$ConfidenceInAgeEstimate>= 90 & dat1$Tissue == "Blood"&
                  dat1$SpeciesCommonName == "California sea lion")

  plotfn(dat1, nc, train.idx, "Sea lion, Blood, LOO",
       includelegend=FALSE, tissuecolor = FALSE)

}
dev.off()

############## Figure 1
if(FALSE){
  plotfn = function(dat1, nc, idx, name1, includelegend=FALSE, tissuecolor = TRUE){
    ylab = strsplit(colnames(dat1)[nc],"_")[[1]][2]
    x = dat1$Age[idx]
    y = dat1[idx, nc]
    xr = range(x,y, na.rm = T)
    tab1 = unique(dat1[idx,]%>%
                select(SpeciesCommonName,SpeciesNumber))%>%
      arrange(SpeciesNumber)

    plot(x, y, type="n", xlab = paste0("Age (N=",length(x),")"),
         ylab = paste(ylab),
         main = paste0(name1,"\n"),
         xlim=xr, ylim=xr,)
    text(x,y, dat1$SpeciesNumber[idx], col = dat1$TissueColor[idx])
    if(includelegend) legend("topleft",paste(tab1[,1], tab1[,2],sep = ": "))
    if(tissuecolor){
      legend("bottomright",c("Blood","Skin"),pch = 19,
           col = c("red","blue"))
    }
    abline(0,1, lty=2)
    abline(coef(lm(y~x)), lwd=2)

  }

}
```

```
colnames(dat1)
unique(dat1%>%select(SpeciesLatinName,SpeciesCommonName,
                   SpeciesNumber))

tiff(paste0(outfolder,"/pinniped_fig1_v6.tiff"),
     width = 800,height = 800)
par(mfrow=c(2,2),mar = c(5, 5, 4, 2) + 0.1)
{

  nc1 = 55
  train.idx = which(dat1$CanBeUsedForAgingStudies=="yes"&
                    dat1$ConfidenceInAgeEstimate>= 90)
  plotfn(dat1, nc1, train.idx, "a. All Pinniped Samples",
       includelegend=FALSE)
  legend("bottomright",c("Blood","Skin"),
       pch = 19, col = c("red","blue"))
  legend("topleft",c("California sea lion: 1",
                   "Harbor seal: 2",
                   "Pacific walrus: 3",
                   "Harp seal: 4",
                   "Australian sea lion: 5",
                   "Steller sea lion: 6"))


  nc=56
  train.idx = which(dat1$CanBeUsedForAgingStudies=="yes"&
                    dat1$ConfidenceInAgeEstimate>= 90 & dat1$Tissue == "Blood")
  plotfn(dat1, nc, train.idx, "b. Blood Samples",
       includelegend=FALSE)
  legend("topleft",c("California sea lion: 1",
                   "Harbor seal: 2",
                   "Pacific walrus: 3",
                   "Harp seal: 4"))

}
{
  nc1 = 63
  train.idx = which(dat1$CanBeUsedForAgingStudies=="yes"&
                    dat1$ConfidenceInAgeEstimate>= 90)
  plotfn(dat1, nc1, train.idx, "c. All Samples, LOSO",
       includelegend=FALSE)
  legend("bottomright",c("Blood","Skin"),
       pch = 19, col = c("red","blue"))
```

```
  legend("topleft",bg="transparent",
       c("California sea lion: 1",
         "Harbor seal: 2",
         "Pacific walrus: 3",
         "Harp seal: 4",
         "Australian sea lion: 5",
         "Steller sea lion: 6"))

  nc1 = 64
  train.idx = which(dat1$CanBeUsedForAgingStudies=="yes"&
                 dat1$ConfidenceInAgeEstimate>= 90 &
                 dat1$Tissue == "Blood")
  plotfn(dat1, nc1, train.idx, "d. Blood Samples, LOSO",
       includelegend=FALSE)
  legend("topleft",bg="transparent",
       c("California sea lion: 1",
         "Harbor seal: 2",
         "Pacific walrus: 3",
         "Harp seal: 4"))

}
dev.off()

tiff(paste0(outfolder,"/pinniped_fig1_v6.tiff"),
    width = 800,height = 800)
par(mfrow=c(2,2),mar = c(5, 5, 4, 2) + 0.1)
{
  nc1 = 60
  train.idx = which(dat1$CanBeUsedForAgingStudies=="yes"&
                 dat1$ConfidenceInAgeEstimate>= 90 &
                 dat1$SpeciesNumber == 1)
  plotfn(dat1, nc1, train.idx, "California Sea Lion",
       includelegend=FALSE)


  nc= 58
  train.idx = which(dat1$CanBeUsedForAgingStudies=="yes"&
                 dat1$ConfidenceInAgeEstimate>= 90 &
                 dat1$Tissue == "Blood"&
                 dat1$SpeciesNumber == 1)
  plotfn(dat1, nc, train.idx, "California Sea Lion Blood",
       includelegend=FALSE,tissuecolor = FALSE)

}
```

```
dev.off()

tiff(paste0(outfolder,"/pinniped_fig3.tiff"),
     width = 800,height = 400)
par(mfrow=c(1,2),mar = c(5, 5, 4, 2) + 0.1)
{
  nc1 = 59
  train.idx = which(dat1$CanBeUsedForAgingStudies=="yes"&
                    dat1$ConfidenceInAgeEstimate>= 90 &
                    dat1$SpeciesNumber == 2)
  plotfn(dat1, nc1, train.idx, "Harbor Seal",
         includelegend=FALSE)


  nc= 57
  train.idx = which(dat1$CanBeUsedForAgingStudies=="yes"&
                    dat1$ConfidenceInAgeEstimate>= 90 &
                    dat1$Tissue == "Blood"&
                    dat1$SpeciesNumber == 2)
  plotfn(dat1, nc, train.idx, "Harbor Seal Blood",
         includelegend=FALSE,tissuecolor = FALSE)

}
dev.off()

tiff(paste0(outfolder,"/pinniped_fig2a.tiff"),
     width = 800,height = 400)
par(mfrow=c(1,2),mar = c(5, 5, 4, 2) + 0.1)
{
  nc1 = 60
  train.idx = which(dat1$CanBeUsedForAgingStudies=="yes"&
                    dat1$ConfidenceInAgeEstimate>= 90 &
                    dat1$SpeciesNumber %in% c(1,5,6))
  plotfn(dat1, nc1, train.idx, "Otariid",
         includelegend=FALSE)
  legend("bottomright",c("Blood","Skin"),
         pch = 19, col = c("red","blue"))
  legend("topleft",c("California sea lion: 1",
                   "Australian sea lion: 5",
                   "Steller sea lion: 6"))


  nc= 58
  train.idx = which(dat1$CanBeUsedForAgingStudies=="yes"&
```

```
                    dat1$ConfidenceInAgeEstimate>= 90 &
                    dat1$Tissue == "Blood"&
                    dat1$SpeciesNumber %in% c(1,5,6))
  plotfn(dat1, nc, train.idx, "California Sea Lion Blood",
         includelegend=FALSE)

}
dev.off()


tiff(paste0(outfolder,"/pinniped_fig3a.tiff"),
     width = 800,height = 400)
par(mfrow=c(1,2),mar = c(5, 5, 4, 2) + 0.1)
{
  nc1 = 59
  train.idx = which(dat1$CanBeUsedForAgingStudies=="yes"&
                    dat1$ConfidenceInAgeEstimate>= 90 &
                    dat1$SpeciesNumber %in% c(2,4))
  plotfn(dat1, nc1, train.idx, "Phocid",
         includelegend=FALSE)
  legend("bottomright",c("Blood","Skin"),
         pch = 19, col = c("red","blue"))
  legend("topleft",c("Harbor seal: 2",
                   "Harp seal: 4"))


  nc= 57
  train.idx = which(dat1$CanBeUsedForAgingStudies=="yes"&
                    dat1$ConfidenceInAgeEstimate>= 90 &
                    dat1$Tissue == "Blood"&
                    dat1$SpeciesNumber %in% c(2,4))
  plotfn(dat1, nc, train.idx, "Phocid Blood",
         includelegend=FALSE)
  legend("topleft",c("Harbor seal: 2",
                   "Harp seal: 4"))

}
dev.off()

######## v6 combined
tiff(paste0(outfolder,"/pinniped_fig2_v6.tiff"),
     width = 800,height = 800)
par(mfrow=c(2,2),mar = c(5, 5, 4, 2) + 0.1)
{
```

```
  nc1 = 59
  train.idx = which(dat1$CanBeUsedForAgingStudies=="yes"&
                  dat1$ConfidenceInAgeEstimate>= 90 &
                  dat1$SpeciesNumber %in% c(2,4))
  plotfn(dat1, nc1, train.idx, "a. Phocid",
       includelegend=FALSE)
  legend("bottomright",c("Blood","Skin"),
       pch = 19, col = c("red","blue"))
  legend("topleft",c("Harbor seal: 2",
                  "Harp seal: 4"))


  nc= 57
  train.idx = which(dat1$CanBeUsedForAgingStudies=="yes"&
                  dat1$ConfidenceInAgeEstimate>= 90 &
                  dat1$Tissue == "Blood"&
                  dat1$SpeciesNumber %in% c(2,4))
  plotfn(dat1, nc, train.idx, "b. Phocid Blood",
       includelegend=FALSE)
  legend("topleft",c("Harbor seal: 2",
                  "Harp seal: 4"))

}
{
  nc1 = 60
  train.idx = which(dat1$CanBeUsedForAgingStudies=="yes"&
                  dat1$ConfidenceInAgeEstimate>= 90 &
                  dat1$SpeciesNumber %in% c(1,5,6))
  plotfn(dat1, nc1, train.idx, "c. Otariid",
       includelegend=FALSE)
  legend("bottomright",c("Blood","Skin"),
       pch = 19, col = c("red","blue"))
  legend("topleft",c("California sea lion: 1",
                  "Australian sea lion: 5",
                  "Steller sea lion: 6"))


  nc= 58
  train.idx = which(dat1$CanBeUsedForAgingStudies=="yes"&
                  dat1$ConfidenceInAgeEstimate>= 90 &
                  dat1$Tissue == "Blood"&
                  dat1$SpeciesNumber %in% c(1,5,6))
  plotfn(dat1, nc, train.idx, "d. California Sea Lion Blood",
       includelegend=FALSE)
```

```
}
dev.off()
```
